# Why still in hospital after fast-track hip and knee arthroplasty?

**DOI:** 10.3109/17453674.2011.636682

**Published:** 2011-11-25

**Authors:** Henrik Husted, Troels H Lunn, Anders Troelsen, Lissi Gaarn-Larsen, Billy B Kristensen, Henrik Kehlet

**Affiliations:** ^1^Department of Orthopaedic Surgery; ^2^Department of Anaesthesiology, Hvidovre University Hospital; ^3^Section of Surgical Pathophysiology, Rigshospitalet, Copenhagen University; ^4^The Lundbeck Centre for Fast-track Hip and Knee Arthroplasty, Copenhagen, Denmark

## Abstract

**Background and purpose:**

Length of stay (LOS) following total hip and knee arthroplasty (THA and TKA) has been reduced to about 3 days in fast-track setups with functional discharge criteria. Earlier studies have identified patient characteristics predicting LOS, but little is known about specific reasons for being hospitalized following fast-track THA and TKA.

**Patients and methods:**

To determine clinical and logistical factors that keep patients in hospital for the first postoperative 24–72 hours, we performed a cohort study of consecutive, unselected patients undergoing unilateral primary THA (n = 98) or TKA (n = 109). Median length of stay was 2 days. Patients were operated with spinal anesthesia and received multimodal analgesia with paracetamol, a COX-2 inhibitor, and gabapentin—with opioid only on request. Fulfillment of functional discharge criteria was assessed twice daily and specified reasons for not allowing discharge were registered.

**Results:**

Pain, dizziness, and general weakness were the main clinical reasons for being hospitalized at 24 and 48 hours postoperatively while nausea, vomiting, confusion, and sedation delayed discharge to a minimal extent. Waiting for blood transfusion (when needed), for start of physiotherapy, and for postoperative radiographic examination delayed discharge in one fifth of the patients.

**Interpretation:**

Future efforts to enhance recovery and reduce length of stay after THA and TKA should focus on analgesia, prevention of orthostatism, and rapid recovery of muscle function.

Total hip and total knee arthroplasty (THA and TKA) are frequent operations with an average length of stay (LOS) of about 6–12 days in the United Kingdom, Germany, and Denmark (Husted et al. 2006, Bundesauswertung 2009, NHS 2010).

During the last decade, however, there has been increased interest in optimal multimodal perioperative care to enhance recovery (the fast-track methodology). Improvement of analgesia; reduction of surgical stress responses and organ dysfunctions including nausea, vomiting, and ileus; early mobilization; and oral nutrition have been of particular interest (Kehlet 2008, Kehlet and Wilmore 2008). These principles have also been applied to THA and TKA, resulting in improvements in pain treatment with multimodal opioid-sparing regimens including a local anesthetic infiltration technique (LIA) or peripheral nerve blocks to facilitate early mobilization (Ilfeld et al. 2006a, b, 2010a, Andersen et al. 2008, Kerr and Kohan 2008), and allowing functional rehabilitation to be initiated a few hours postoperatively (Holm et al. 2010)—ultimately leading to a reduction in LOS (Husted et al. 2008, Barbieri et al. 2009, Husted et al. 2010a, b). Using these evidence-based regimens combined with an improved logistical setup, LOS is reduced to about 2–4 days (Kerr and Kohan 2008, Husted et al. 2010 a,b,c, Lunn et al. 2011).

Having well-defined functional discharge criteria is imperative in order to ensure a safe discharge—and it is mandatory if meaningful comparison of LOS is done following alterations in the track (Husted et al. 2008). In the same fast-track setting, an earlier study focused on patient characteristics predicting LOS (Husted et al. 2008). However, little is known about the specific reasons for why patients are hospitalized during the first 1–3 days after THA or TKA; i.e. why can patients not be discharged?

We therefore analyzed clinical and organizational factors responsible for being hospitalized in a well-defined prospective setup in a fast-track unit. This unit had previously documented LOS of about 2–3 days (Andersen et al. 2008, Holm et al. 2010, Husted et al. 2010b, c, Lunn et al. 2011).

## Patients and methods

According to Danish law, this quality-assurance study did not require approval by an ethics committee. It was registered at ClinicalTrials.gov (NTC01047371).

A well-defined fast track was implemented at the Orthopedics Department of Hvidovre University Hospital in 2003. Since then, all patients undergoing THA and TKA have been enrolled in the program. The fast-track setup has optimized logistical and evidence-based clinical features (Husted et al. 2008). All patients attend a multidisciplinary preoperative patient seminar for information—including discharge criteria and mention of intended LOS of 1–3 days—and encouragement. No patient is excluded from the fast-track setup; every patient is included regardless of age, co-morbidities, ASA score, living situation etc.

Patients undergo surgery on Monday, Tuesday, or Wednesday, as the ward is a 5-day unit and is closed at weekends. Treatment of pain and early mobilization have been of particular interest (Andersen et al. 2008, Holm et al. 2010). Patients are operated under spinal anesthesia with 1.5–2.5 mL 0.5% (7.5–12.5 mg) hyperbaric or plain bupivacaine (depending on procedure and expected surgery time) administered via the L2/L3 or L3/L4 vertebral interspace with a standardized intraoperative regime for fluid administration, consisting of 0.9% saline (5 mL/kg/h) and colloid (Voluven; 7.5 mL/kg/h) (Holte et al. 2007). Also, a standardized program in the operating theater is followed, including use of tranexamic acid (1 g) and no use of drains. Patients are operated using a posterior approach for THA and with a standard midline skin incision and a medial parapatellar approach for TKA. Patients who undergo TKA have local analgesics infiltrated into the soft tissues (LIA) using a systematic technique (Andersen et al. 2008, Kerr and Kohan 2008).

Postoperatively, patients are transferred to the post-anesthesia care unit (PACU) and then to a specialized knee and hip arthroplasty unit with an implemented, well-defined multimodal fast-track rehabilitation regime (Husted et al. 2008). They are discharged from the PACU after 1–2 h and encouraged to ambulate upon arrival at the ward. Physiotherapy is started within the first 24 h and takes place once or twice daily until discharge. Physiotherapy concentrates on range of motion of the operated joint, strengthening of the muscles, and gain of a normal gait pattern with crutches. Multimodal oral opioid-sparing analgesia is given to all patients (Cox2 inhibitor (celecoxib; 200 mg/12 hourly) paracetamol (slow release: 2 g/12 hourly), gabapentin (300 mg morning and 600 mg evening), with opioid only upon request). For thromboprophylaxis, orally administered Xarelto (rivaroxaban; 10 mg) is administered once a day until discharge, starting 6–8 h postoperatively.

LOS is counted as the number of postoperative nights in hospital until discharge. Strictly functional discharge criteria are applied (ability to get dressed independently, ability to get in and out of bed, ability to sit and rise from a chair/toilet, independence in personal care, mobilization with walker/crutches, and ability to walk > 70 m with crutches). In addition, sufficient oral pain treatment (VAS < 5 on activity) and acceptance of discharge are prerequisites for discharge. All patients are discharged directly to their homes.

The following preoperative parameters were registered: type of operation, age, sex, preoperative diagnosis, and weekday of operation. The following outcome parameters were registered: LOS, fulfillment of each of the discharge criteria, and detailed reason(s) for not being discharged, as assessed twice daily—at 9 a.m. and at 2 p.m. (Tables 1 and 2).

**Table 1.** Fulfillment of functional discharge criteria at 9 a.m. and 2 p.m. on various days. The accumulated proportions of patients not discharged are shown at the top of the table, as is their median age and gender distribution. Below, proportions of patients fulfilling the specified discharge criteria are shown (as number of patients fulfilling each one divided by the number of patients remaining in hospital)

	Op-day	Day 1	Day 1	Day 2	Day 2	Day 3	Day 3
Evaluation	2 p.m.	9 a.m.	2 p.m.	9 a.m.	2 p.m.	9 a.m.	2 p.m.
Not discharged							
TKA	100%	94%	80%	33%	27%	7%	5%
THA	100%	87%	60%	22%	20%	9%	6%
Age and gender **^a^**							
TKA	68 (48/61)	69 (45/57)	70 (40/47)	68 (16/20)	70 (13/11)	66 (6/2)	61 (4/1)
THA	65 (40/58)	65 (34/51)	66 (22/36)	70 (9/13)	77 (5/10)	80 (3/6)	82 (2/4)
Clothes							
TKA	62% **^c^**	56%	75%	86%	75%	77%	67%
THA	44% **^c^**	57%	76%	90%	77%	81%	67%
Bed							
TKA	56%	64%	80%	94%	83%	91%	83%
THA	48%	61%	79%	90%	77%	88%	67%
Chair							
TKA	60%	70%	87%	95%	81%	86%	83%
THA	51%	77%	87%	93%	77%	88%	89%
Care							
TKA	17%	62%	78%	92%	81%	77%	67%
THA	9%	63%	84%	92%	77%	88%	78%
Mobilized							
TKA	62%	74%	85%	96%	86%	86%	83%
THA	60%	81%	92%	93%	82%	88%	78%
Walking 70 m **^b^**							
THA	6%	14% **^c^**	44%	68%	72%	77%	50%
TKA	4%	27% **^c^**	54%	80%	64%	56%	56%

**^a^** Median age and gender distribution (number of men/women)

**^b^** with crutches

**^c^** Significant difference between TKA and THA

**Table 2.** Reasons for patients not being able to be discharged at 9 a.m. and 2 p.m. on various days. The accumulated proportions of patients not discharged are shown at the top of the table. Below that, reasons for not fulfilling the specified discharge criteria are shown (as number of patients with each clinical problem divided by the number of patients remaining in hospital)

	Op-day	Day 1	Day 1	Day 2	Day 2	Day 3	Day 3
Evaluation	2 p.m.	9 a.m.	2 p.m.	9 a.m.	2 p.m.	9 a.m.	2 p.m.
Not discharged							
TKA	100%	94%	80%	33%	27%	7%	5%
THA	100%	87%	60%	22%	20%	9%	6%
Pain **^a^**							
TKA	53%	43% **^b^**	29%	19%	0% **^b^**	0%	0%
THA	47%	24% **^b^**	22%	18%	20% **^b^**	11%	0%
Dizziness							
TKA	11%	24%	15%	17%	17%	13%	0%
THA	15%	21%	17%	14%	20%	11%	0%
PONV **^c^**							
TKA	13%	8%	7%	3%	7%	0%	0%
THA	11%	5%	5%	5%	5%	0%	0%
Confusion							
TKA	1%	0%	0%	3%	4%	0%	0%
THA	2%	2%	2%	0%	0%	0%	0%
Sedation							
TKA	3%	5%	6%	6%	4%	13%	20%
THA	1%	7%	5%	5%	0%	0%	0%
Muscle weakness **^d^**							
TKA	16% **^b^**	18%	13% **^b^**	25%	8%	13%	20%
THA	29% **^b^**	28%	26% **^b^**	18%	13%	44%	17%
Technical **^e^**							
TKA	16%	10%	2%	0%	8%	13%	20%
THA	15%	12%	9%	5%	0%	0%	0%
“Logistics” **^f^**							
TKA	22%	27%	20%	33%	21%	25%	40%
THA	18%	35%	20%	36%	20%	44%	50%

**^a^** Pain > 5 with activity

**^b^** Significant difference between TKA and THA

**^c^** Postoperative nausea and vomiting

**^d^** or lack of sufficient control to ambulate

**^e^** Ongoing intravenous transfusion of blood or plasma expander, or urinary catheter due to urinary retention

**^f^** Waiting for physiotherapy or postoperative radiographs

### Statistics

Data are presented as median values with interquartile range (IQR) and range, or as proportions given as a percentage value. Data that are not normally distributed were compared using a 2-sample Wilcoxon rank-sum (Mann-Whitney) test. Proportions were compared using the Pearson chi-square test or (for small sample sizes) Fisher's exact test. Median age and sex distribution for the patients not fulfilling discharge criteria are presented for the various time points for evaluation. Analyses were performed using the STATA version 10.1 software (StataCorp LP, College Station, TX) and p-values of < 0.05 were considered significant.

## Results

We included 215 consecutive unselected patients who underwent primary TKA or THA from January 2010 to June 2010. 8 registration forms had not been filled in completely, leaving 207 patients (96%) for final evaluation.

109 patients underwent TKA and 98 underwent THA (117 women in total). Mean age was 66 (21–94) years. Length of stay (LOS) was median 2 (IQR: 1–2; range: 1–18) for TKA and median 2 (IQR: 1–2, range: 1–11) for THA. Mean LOS was 2.4 days for TKA and 2.2 days for THA. There was no difference regarding LOS for patients who were operated on Monday, Tuesday, or Wednesday (p > 0.3), and 196 patients (95%) were discharged no later than 2 p.m. on day 3 (72 h postoperatively).

Table 1 shows fulfillment of functional discharge criteria at 9 a.m. and 2 p.m. on various days. There was no difference between TKA and THA, except a better ability to get dressed independently at 2 p.m. on the day of surgery in favor of TKA (p = 0.01) and better ability to walk 70 meters at 9 a.m. on day 1 in favor of THA (p = 0.02). Apart from difficulties in performing personal care and walking with crutches 70 m at 2 p.m. on the day of surgery, all criteria were mastered approximately at the same time even though some patients were operated as first cases while others were second or third cases. For TKA, LOS was not related to age, but for THA the few patients who were not discharged after > 2 days were older than the ones discharged < 2 days (Table 1).

Table 2 shows specific underlying reasons for patients not being able to be discharged at 9 a.m. and 2 p.m. on various days. Pain, dizziness, and muscle weakness were the main clinical reasons for delaying discharge. Postoperative nausea and vomiting on the day of operation and on the first postoperative day was a small problem (< 13%), and confusion and sedation was an even smaller problem (< 7%). Technical reasons (ongoing intravenous transfusion of blood or plasma expander, or urinary catheter due to urinary retention) were only an issue for patients in the first 24 hours. “Logistics” (waiting for physiotherapy, postoperative radiographs, etc.) was a challenge from operation until discharge for at least one fifth of patients. There was a difference between TKA and THA regarding pain only at 9 a.m. on day 1 and at 2 p.m. on day 2 (p = 0.01 and p = 0.05, respectively). In addition, at 2 p.m. on the day of operation and at 2 p.m. on day 1 there was a difference between TKA and THA regarding muscle weakness (p = 0.02 and p = 0.04, respectively).

Although they fulfilled the functional discharge criteria (Table 1), some patients were not discharged immediately thereafter (Table 2 and Figure 1). Pain (> 5 on VAS scale for activity), logistical reasons (waiting for blood transfusion, for physiotherapy, and for postoperative radiographs), and patient-related factors (feeling insecure) were responsible for this delay. Thus, for these reasons, 6 patients (3%) fulfilled the functional discharge criteria at 2 p.m. on the day of surgery without being subsequently discharged.

**Figure:**
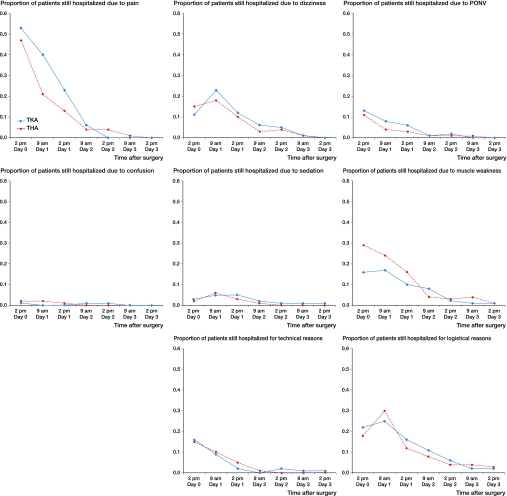
Proportion of patients of the 2 cohorts (TKA, n = 107; THA, n = 98) still hospitalized during the first 72 hours, and reasons. Proportions are calculated as number of patients with each clinical problem divided by the total number of TKAs and THAs, respectively. PONV: postoperative nausea and vomiting. Technical reasons cover ongoing intravenous transfusion of blood or plasma expander, or urinary catheter due to urinary retention. “Logistical” covers waiting for physiotherapy or postoperative radiographs.

## Discussion

Our findings offer the possibility of safe reduction of LOS after fast-track THA and TKA. We have previously shown that our fast-track regime for THA and TKA does not increase re-admissions because of complications (Husted et al. 2010c, d). The main finding of our study, in which almost four-fifths of patients could be discharged within 48 hours postoperatively—and nearly all within 72 hours—was that pain, dizziness, and general weakness were responsible for not being discharged earlier in about 80% of patients while organizational factors were a reason in about 20% of patients. This was in spite of the fact that the fast-track unit has had a long period of development (Husted et al. 2008). Although nausea, vomiting, sedation, and confusion had little influence on the need for hospitalization, they may be reduced further by more intensive multimodal non-opioid pain management (Lunn et al. 2011).

Almost half of the patients fulfilled the 6 well-defined functional discharge criteria in the afternoon on the day of the operation. With the others, the main problems were personal care and walking 70 meters with crutches. This may call for reconsideration regarding instant access to physiotherapy and further improvement of analgesia. Analgesia may be improved with continuous peripheral nerve blocks (Ilfeld et al. 2006a,b, 2008, 2010a) but the drawback is a risk of muscle weakness, a need for adjustment of infusion dose of local anesthetics, and risk of falls (Kandasami et al. 2009, Ilfeld et al. 2010b, Sharma et al. 2010).

Optimization of analgesia may include a high dose of glucocorticoids preoperatively (Lunn et al. 2011) or use of other non-opioid analgesics such as gabapentinoids (Tiippana et al. 2007).

Since dizziness and muscle weakness were other main reasons for delayed discharge, prevention of orthostatic hypotension (Bundgaard-Nielsen et al. 2009), which may contribute to the early dizziness associated with mobilization, should be of interest. As muscle weakness also appears to be a problem in the early postoperative period, the reduced quadriceps muscle function amounting to about 60–80% reduction after TKA (Mizner et al. 2005, Holm et al. 2010) and to about 30–40% after THA (Holm et al. 2011), may call for early physiotherapy including strengthening exercises and/or reduction of inhibitory neural reflexes that may contribute to impaired muscle function (Mizner et al. 2005).

In other studies, short hospital stays of 1–2 days have been achieved in selected patients, but no specific information was provided on potential discharge problems (Ilfeld et al. 2006a,b, Kerr and Kohan 2008), except in one study (Berger et al. 2009). In this latter study on THA patients only, discharge criteria were similar to ours, but patients were highly selected based on age, co-morbidities, BMI, case number on day of surgery etc. Furthermore, problems delaying discharge were not described in detail and patients also had home-based nursing and physiotherapy. It should be emphasized that in our study, all the patients were discharged to their homes and not to a secondary rehabilitation unit, as we had previously found that the fast-track setup does not result in more re-admissions, more thromboembolic episodes, more home-based support, or more use of physiotherapy or general practitioners (Husted et al. 2010 a,c,d).

One of the reasons for our successful program may be that operations are performed in the first 3 days of the week. We have previously found that operations on Thursdays or Fridays may lead to longer stays, due to the upcoming weekend, with a resulting decrease in activities including physiotherapy and surgical rounds (Husted et al. 2008). Finally, improvement of organizational issues such as planning of postoperative radiographs to be taken on the day of surgery or on the first postoperative morning may also facilitate earlier discharge.

There was a tendency for patients discharged > 2 days to be older than patients discharged < 2 days, as previously reported (Husted et al. 2008).

In conclusion, we found that almost all unselected THA and TKA patients can be discharged within 3 days of surgery. Reduction of this time to 1–2 days may be achieved by improvement of perioperative analgesia (multimodal, non-opioid), reduction of the risk of orthostatic hypotension, improvement of quadriceps muscle function, and avoidance of logistical problems hindering early discharge.
